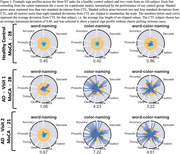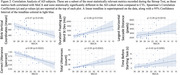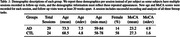# Multimodal Analysis of Behavior During Stroop Test for Characterization of Alzheimer’s Disease Signs

**DOI:** 10.1002/alz.094305

**Published:** 2025-01-09

**Authors:** Trevor Meyer, Anna Favaro, Esther S Oh, Pedro Irazoqui, Najim Dehak, Laureano Moro‐Velazquez

**Affiliations:** ^1^ Johns Hopkins University, Baltimore, MD USA; ^2^ Johns Hopkins University School of Medicine, Baltimore, MD USA

## Abstract

**Background:**

Clinical rating scales and neuropsychological tests are commonly used for assessing sign and disease severity, yet lack detail in the early stages Alzheimer’s Disease (AD). Existing evaluation methods can be subjective, nonlinear, expensive, or reliant on anecdotal evidence making objective and consistent characterization and phenotyping of AD difficult. Multimodal analysis of patient behavior, rather than scoring of patient‐generated output which can be skewed by compensation strategies, presents a unique opportunity to objectively quantify AD related changes. Using the Stroop Test (ST), we present a multimodal analysis of eye movement and spoken responses during completion of ST tasks, with the goal to characterize patient behavior.

**Method:**

17 individual AD subjects and 24 age‐matched cognitively normal controls (CTL) were recruited from the Johns Hopkins Memory and Alzheimer’s Treatment Center and the Movement Disorders Clinic at the Johns Hopkins University School of Medicine. Eight subjects returned for two or three follow up sessions at least 20 weeks apart, totaling 29 AD recording sessions. An Eyelink Portable Duo in head‐free‐to‐move mode was used to record eye movements and spoken responses. We analyzed subjects' interaction with ST using several metrics relating to blinking, saccade, fixation, gaze, prosodic, accuracy, and timing characteristics. We summarize patient behavior abnormality by scaling behavioral metrics into positive z‐scores using CTL performance and summarize the overall abnormality as the average z‐score across 45 metrics.

**Result:**

The average deviation across metrics correlates with MoCA scores (p<0.001). These metrics display significantly different distributions between the AD from CTL subjects (p<0.001). A visualization we've coined as a “sign profile” summarizes patient behavior abnormality during ST. In all subjects our abnormality measurement closely matches MoCA scores and clinician observations.

**Conclusion:**

AD subjects execute the ST with eye movement and speech behaviors which significantly differ from CTL subjects, and subjects with longitudinal recordings show encouraging results for the use of this analysis in monitoring. By utilizing measures from more than one feature, we detect early clinical changes a single‐score test might miss. This analysis offers comprehensive patient profiling, providing an intuitive and quantitative understanding of patient signs enabling more accurate detection and management of disease.